# Concealing, tolerating, and adjusting to emotions in obsessive-compulsive and anxiety disorders: a cross-sectional study

**DOI:** 10.47626/2237-6089-2023-0652

**Published:** 2025-02-27

**Authors:** Carla P. Loureiro, Emma M. Thompson, Luana D. Laurito, Maria E. Moreira-de-Oliveira, Rafaela V. Dias, Gabriela B de Menezes, Leonardo F. Fontenelle

**Affiliations:** 1 Programa de Ansiedade, Obsessões e Compulsões Instituto de Psiquiatria Universidade Federal do Rio de Janeiro Rio de Janeiro RJ Brazil Programa de Ansiedade, Obsessões e Compulsões, Instituto de Psiquiatria, Universidade Federal do Rio de Janeiro (UFRJ), Rio de Janeiro, RJ, Brazil.; 2 Turner Institute for Brain and Mental Health Monash University Melbourne VIC Australia Turner Institute for Brain and Mental Health, Monash University, Melbourne, VIC, Australia.; 3 Instituto D’Or de Pesquisa e Ensino Rio de Janeiro RJ Brazil Instituto D’Or de Pesquisa e Ensino, Rio de Janeiro, RJ, Brazil.

**Keywords:** Affective style, ASQ, emotion regulation, OCD, anxiety disorders

## Abstract

**Objective:**

Although research has shown that mood and anxiety disorders manifest disturbed emotion regulation, it is unclear whether anxiety disorders differ from each other in terms of their emotion regulation strategies. In the present study, we investigated whether patients with anxiety disorders present different affective styles.

**Methods:**

We assessed the affective styles of 32 obsessive-compulsive disorder (OCD) patients, 29 social anxiety disorder (SAD) patients, 29 panic disorder (PD) patients, and 20 healthy controls using the Affective Style Questionnaire (ASQ). A multivariate analysis of covariance (MANCOVA) was conducted to compare affective styles across groups (OCD, SAD, PD, and control), while controlling for depression, anxiety symptoms, and age.

**Results:**

The MANCOVA revealed a significant, small-medium, main effect of diagnostic group on affective styles. The planned contrasts revealed that OCD and SAD patients reported significantly lower scores for “tolerance” (ASQ-T) compared to the healthy controls. There were no differences between the PD group and healthy controls.

**Conclusion:**

Our findings provide evidence that individuals with OCD and SAD have difficulty tolerating strong emotions existing in the present moment in an open and non-defensive way.

## Introduction

Emotions may be experienced uniquely by different individuals, whether in terms of type and/or intensity, even when faced with the same stimulus. Emotional regulation is a human being’s ability to, consciously or otherwise, influence their emotional experiences, such as intensity and expression, to respond appropriately to an environmental demand.^1,2^ Individuals use different regulation strategies to modify emotions, especially those with negative valences. These differences in affective experiences and preferences for certain strategies to cope with emotions are called “affective styles.”^3^ Hoffmann and Kashdan^1^ developed the Affective Style Questionnaire (ASQ) for assessing different affective styles. The ASQ comprises a 20-item Likert scale with three subscales: concealing (ASQ-C), adjustment (ASQ-A), and tolerance (ASQ-T).

According to Hoffman and Kashdan,^1^ concealing encompasses suppression of an emotion along with other strategies aiming to hide or avoid such emotions once they are fully activated (e.g., item 1- “People usually can’t tell how I’m feeling inside”). Adjustment includes modulation of negative emotions according to contextual demands, effectively balancing and adapting emotional experiences and expressions as needed (e.g., item 4- “I can avoid getting upset by trying to see things from another perspective”). The third style, tolerance, refers to strategies focused on experiencing emotions that exist in the present moment in a non-defensive and open way. This style, which includes acceptance and mindfulness strategies, allows one to tolerate strong emotions without attempting to modify or reduce emotional experiences (e.g., item 11- “It’s okay to sometimes have negative emotions”).

Validation studies of the ASQ have pointed to a possible association between affective styles and mental disorders.^4-6^ For instance, the “adjustment” affective style seems to be negatively associated with symptoms of depression, stress, and anxiety within a clinical sample.^4-6^ This suggests that individuals suffering from mood and anxiety disorders would tend to have greater difficulty in adjusting negative affect according to situational demands. In turn, the “concealing” affective style showed a positive association with anxiety, depression, and stress,^5,6^ suggesting that emotion suppression is a detrimental strategy for alleviating subjective distress in people with anxiety and mood disorders.^7^ The “tolerance” affective style showed a negative relationship with stress and anxiety.^5,6^

Since previous studies have focused on multiple but mixed categories of anxiety and depressive disorders, it remains unclear whether anxiety disorders differ from each other in terms of affective style. There is some evidence that affective styles may differ between patients suffering from mood and anxiety disorders, since tolerance showed a negative association with anxiety symptoms in patients with mood disorders but not in patients with anxiety disorders.^5^ In the present study, we hypothesized that patients with anxiety disorders have different affective styles. More specifically, we predicted that (a) social anxiety disorder (SAD) patients would be more likely to conceal their emotions, (b) panic disorder (PD) patients would be less tolerant to strong affect, and (c) obsessive-compulsive disorder (OCD) patients would be less likely to adjust their emotions to different contexts (rigidity).

## Methods

### Participants

Participants consisted of OCD (n = 32), SAD (n = 29), or PD (n = 29) patients or individuals with no diagnosis (n = 20), according to the Mini International Neuropsychiatric Interview (MINI). Patients were recruited from those seeking treatment at the anxiety, obsessions, and compulsions program (Programa de Ansiedade, Obsessões e Compulsões) and the panic and breathing laboratory (Laboratório de Pânico e Respiração) run by the Universidade Federal do Rio de Janeiro (UFRJ) Institute of Psychiatry (Instituto de Psiquiatria, IPUB), in Rio de Janeiro, RJ, Brazil. Participants were included if they a) either had a primary diagnosis of OCD, SAD or PD according to Diagnostic and Statistical Manual of Mental Disorders, 4th edition (DSM-IV) criteria or did not meet the criteria for any diagnosis according to the MINI, b) were between 18 and 70 years of age, and c) had sufficient reading and writing ability. Participants exhibiting comorbidity of OCD, SAD, and/or PD were assigned to the group corresponding to their most clinically significant disorder.

Patients with OCD, SAD, or PD were excluded if they also exhibited severe psychiatric illnesses such as dementia, an intellectual disability, or current manic or psychotic episodes. Most patients (N_OCD_ = 28, N_SAD_ = 26, and N_PD_ = 29) were using serotonin reuptake inhibitors, tricyclics, or venlafaxine, among others. A smaller subset of patients was undergoing concomitant psychotherapy (N_OCD_ = 12, N_SAD_ = 6, and N_PD_ = 3).

### Ethical considerations

Participants were first informed about the nature and aims of the study and subsequently provided written consent to participation. They then completed a range of self-report questionnaires in the presence of a psychologist. The research protocol was approved by the IPUB/UFRJ ethics committee (CAAE 50308015.1.0000.5263).

### Assessment

To measure symptom severity, self-report responses were obtained from participants after initial diagnosis with the MINI interview.^8^ Brazilian Portuguese versions of the following self-report measures were used.

#### The Dimensional Obsessive-Compulsive Disorder Scale (DOCS)9

The DOCS is a 20-item self-report measure that assesses the severity of four empirically supported dimensions of OCD: contamination, responsibility for harm, symmetry/incompleteness, and unacceptable thoughts. For each dimension, five items are rated from 0-4, assessing a) time occupied by the obsession/compulsion, b) avoidance, c) distress, d) interference, and e) ability to refrain from or disregard obsessions/compulsions. Total scores range from 0-80, with higher scores indicating greater severity of obsessive-compulsive symptoms. The DOCS has shown a good factor structure, internal consistency, and convergent and divergent validity in clinical and non-clinical samples.^9^

#### The Panic and Agoraphobia Scale (PAS)10

The PAS is a 13-item self-report questionnaire that assesses the severity of panic disorder and agoraphobia. Each item is measured on a five-point Likert scale from 0-4. The scale is based on criteria from the DSM-IV and includes five subscales (panic attacks, agoraphobia and avoidance behaviors, anticipatory anxiety, disability, and worries about health). Total scores are obtained by summing all item scores (range: 0-52) with higher scores indicating more severe panic disorder or agoraphobia. The PAS has shown high internal consistency (α = 0.88), high construct validity,^10^ and good discriminant validity from measures of generalized anxiety and agoraphobia.^10^

#### The Social Phobia Inventory (SPIN)11

The SPIN is a 17-item self-report scale that assesses the presence and severity of social anxiety. The scale has items from each dimension of social anxiety, including fear, avoidance, and physiological arousal. Items are rated on a five-point Likert scale from 0 (“not at all”) to 4 (“extremely”), summing to a total score ranging from 0 to 68. The Portuguese SPIN has shown acceptable internal consistency (α = 0.63-0.90) and concurrent validity with other social phobia scales.^12^

#### The Beck Anxiety Inventory (BAI)13

The BAI is a widely used 21-item self-report scale that measures anxiety severity. Each item represents a common symptom of anxiety, and respondents are asked to rate which symptoms they have experienced in the past month from 0 (“not at all”) to 3 (“severely – it bothered me a lot”). Total scores are calculated by summing all responses (range: 0-63). The Portuguese version of the BAI has shown adequate internal reliability and good convergent validity with other anxiety measures.^14^

#### Beck Depression Inventory (BDI )15

The BDI is a 21-item measure of depressive symptom severity based on the key DSM-IV criteria for major depression. Each item is rated from 0 to 3 to reflect the intensity of symptoms the respondent has experienced during the past week. Items are summed to obtain a total score between 0 and 63. The Portuguese version of the BDI has demonstrated high internal consistency (α = 0.81-0.88) and convergent validity.^16^

#### The Affective Style Questionnaire (ASQ)1

The ASQ is a 20-item self-report questionnaire that measures individual differences in sensitivity to and regulation of emotions. The questionnaire is made up of three subscales representing different affective styles: Concealing (attempts to conceal or suppress affect [eight items]); Adjusting (ability to adjust, manage, and work with emotions when needed [seven items]); and Tolerating (an accepting and tolerant attitude towards emotions [five items]). Items are rated from 1 (“not true of me at all”) to 5 (“extremely true of me”) and scores are summed for each subscale. The scale shows acceptable internal consistency (α = 0.65-0.89) and inter-correlations with other measures of emotions regulation, personality, and psychological flexibility support appropriate convergent and discriminant validity.^1^

## Statistical analyses

One-way analysis of variance tests and chi-square tests were used to examine differences in sociodemographic characteristics across diagnostic groups. Normality of residuals and homoscedasticity was confirmed in all ASQ subscales upon inspection of the Shapiro-Wilk test of normality, relevant histograms, and scatterplots. No collinearity was identified, as indicated by the variance inflation factor < 0.10.

 To test our hypotheses, a multivariate analysis of covariance (MANCOVA) was performed comparing affective styles across the diagnostic groups, while controlling for differences in depression and anxiety. Simple comparisons between the control group and the diagnostic groups were planned if the multivariate and between-subjects analyses revealed that one or more ASQ subscale had a significant main effect. Given the exploratory nature of this study, the level of statistical significance was set at 0.05 for all analyses.

## Results

### Descriptive analyses

After removal of two particularly influential outliers, the total sample comprised 110 participants. Sociodemographic characteristics are presented in Table 1. The only statistically significant difference between diagnostic groups was age. Therefore, age was added as a covariate to all analyses.

**Table 1:** Sociodemographic characteristics of the sample and across diagnostic subgroups

	OCD (n = 32)	SAD (n = 29)	PD (n = 29)	HC (n = 20)	Statistics
Age, mean (SD)	38.28 (12.40)	42.07 (14.99)	44.41 (12.05)	29.90 (5.77)	F(3,109) = 6.21; p < 0.001*
Gender (female), n (%)	15 (46.9)	13 (44.8)	18 (62.1)	15 (75.0)	χ^2^(3) = 5.88.27; p = 0.12
Marital status, n (%)					χ^2^(12) = 16.62; p = 0.16
Single	21 (65.6)	19 (65.5)	12 (41.4)	13 (65.0)	
Married	9 (28.1)	7 (24.1)	9 (31.0)	7 (35.0)	
Separated	1 (3.1)	2 (6.9)	6 (20.7)	0.0	
Widowed	0.0	1 (3.4)	2 (6.9)	0.0	
Other	1 (3.1)	0.0	0.0	0.0	
					
Education, n (%)					χ^2^(12) = 9.48; p = 0.66
Less than primary	1 (3.1)	1 (3.4)	4 (13.8)	1 (5.0)	
Primary	2 (6.3)	1 (3.4)	4 (13.8)	0.0	
High school	15 (46.9)	14 (48.3)	11 (37.9)	8 (16.7)	
Tertiary	10 (31.3)	10 (34.5)	7 (24.1)	8 (40.0)	
Postgraduate	4 (12.5)	3 (10.3)	3 (10.3)	3 (15.0)	
					
Ethnicity, n (%)					χ^2^(9) = 9.059; p = 0.043*
White	19 (59.4)	14 (48.3)	17 (58.6)	14 (70.0)	
Black	4 (12.5)	3 (10.3)	3 (10.3)	1 (5.0)	
East Asian	0.0	0.0	2 (6.9)	0.0	
Mixed	9 (28.1)	12 (41.4)	7 (24.1)	5 (25.0)	
					
Currently seeing a psychologist, n (%)	12 (37.5)	6 (20.6)	3 (10.3)	-	χ^2^(2) = 6.28; p = 0.043*
Taking psychotropics, n (%)	28 (87.5)	26 (89.6)	29 (100.0)	-	χ^2^(2) = 2.78; p = 0.249

HC = healthy controls; OCD = obsessive-compulsive disorder; PD = panic disorder; SAD = social anxiety disorder.

* p < 0.05.

Of participants, 17.3% reported a family history of PD, 11.8% reported a family history of SAD, 6.4% reported a family history of OCD, and 41.8% reported a family history of another psychiatric disorder. All other clinical characteristics are presented in Table 2. As shown, the sample exhibited “mild to moderate” depression^15^ and “mild” anxiety.^13^ As expected in a mostly clinical sample, the concealing affective style was the most common affective style.^17^

**Table 2:** Clinical features of final sample, including means and SD for the whole sample and across diagnostic subgroups

	OCD (n = 32)	SAD (n = 29)	PD (n = 29)	HC(n = 20)	Statistics	Post-hoc
	Mean (SD)	Mean (SD)	Mean (SD)	Mean (SD)		
BDI	13.91(8.76)	12.86 (11.52)	15.14 (11.68)	5.60 (5.10)	F(3,106) = 4.14; p = 0.008*	OCD = SAD = PD > HC
BAI	13.16 (12.22)	13.66 (11.79)	25.10 (16.30)	5.25 (6.11)	F(3,106) = 10.64; p < 0.001*	OCD = SAD = HC < PD
PAS	4.59 (8.30)	7.28 (11.30)	13.97 (12.22)	0.85 (2.34)	F(3,106) = 0.8.30; p < 0001*	OCD = SAD = HC < PD
DOCS	22.09 (14.97)	11.21 (11.36)	20.55 (15.32)	5.65 (6.55)	F(3,106) = 9.04; p < 0.001*	OCD = PD > SAD = HC
SPIN	17.59 (17.03)	29.52 (20.33)	21.10 (16.74)	7.55 (8.12)	F(3,106) = 7.01; p < 0.001*	OCD = HC < SAD
ASQ-C	19.28 (5.58)	21.48 (7.42)	21.18 (7.05)	17.95 (5.50)	F(3,105) = 1.60; p = 0.195	OCD = SAD = PD = HC
ASQ-A	17.84 (4.89)	17.21 (3.76)	20.10 (5.54)	20.50 (4.77)	F(3,106) = 3.05; p = 0 .03*	OCD = SAD = PD = HC
ASQ-T	13.50 (3.08)	13.29 (3.81)	14.86 (3.64)	16.65 (3.52)	F(3,104) = 4.54; p = 0.005*	OCD = SAD < HC

ASQ-A = Affective Style Questionnaire, Adjustment subscale; ASQ-C = Affective Style Questionnaire, Concealed subscale; ASQ-T = Affective Style Questionnaire, Tolerance subscale; BAI = Beck Anxiety Inventory; BDI = Beck Depression Inventory; DOCS = Dimensional Obsessive-Compulsive Scale; PAS = Panic and Agoraphobia Scale; SD = standard deviation; SPIN = Social Phobia Inventory.

* p < 0.05.

Regarding affective styles, statistically significant differences were observed between diagnostic groups, specifically for ASQ-A (F[3,106] = 3.05, p = 0.03) and ASQ-T (F[3,104] = 4.54, p = 0.005) scores. Tukey post-hoc testing demonstrated that there was no difference between groups for ASQ-A. The OCD and SAD diagnostic groups had lower scores than healthy controls for ASQ-T, as shown in Table 2.

### MANCOVA

The MANCOVA (n = 109) revealed a significant, small-medium, main effect of diagnostic group on affective styles, after controlling for age, depression, anxiety and undergoing psychotherapy (F[9,288.9] = 2.29, p = 0.017, Λ = 0.81, η_p_^2^ = 0.068). Between-subjects effects showed that only ASQ-T scores differed between diagnostic groups (F[3, 44.5] = 3.84, p = 0.012, η_p_^2^ = 0.107). Planned comparisons revealed that OCD (p = 0.014, 95% confidence interval [95%CI] [-4.96 to -0.58]) and SAD (p = 0.011, 95%CI [-5.00 to -0.659]) diagnostic groups scored significantly lower for ASQ-T than healthy controls.

## Discussion

The purpose of this study was to investigate whether Hofmann and Kashdan’s^1^ affective styles (concealing, adjusting, and tolerating) differ between OCD, SAD, and PD patients and healthy controls. We predicted that SAD patients would be more likely to conceal their emotions, that PD patients would be more intolerant of their emotions, and that OCD patients would be less likely to adjust to new emotions. Although we were unable to confirm these initial hypotheses, we found that both OCD and SAD diagnostic groups were less tolerant of emotions than healthy controls, but also that they did not differ significantly from each other.

Despite reporting findings that were at odds with our initial predictions, our paper adds to previous literature suggesting “experiential avoidance,”^18,19^ or lack of tolerance, is present both in OCD and in SAD.^20,21^ Our results expand the findings linking decreased tolerance to severity of anxiety,^6^ showing it to be particularly relevant in anxiety disorders that, according to the Gray and McNaughton model,^22^ are more clearly characterized by avoidable (i.e., OCD and SAD), rather than unavoidable (i.e., PD) threats.

Gray and McNaughton^22^ propose a taxonomy that classifies anxiety disorders according to two types of threatening stimuli: the avoidable and the unavoidable. According to their model, unavoidable threat stimuli in individuals with PD leads to inhibition of active coping strategies and conservation of resources. We suggest that, lacking active coping strategies during a panic attack, patients with PD would be expected to experience symptoms less defensively. In contrast, in OCD and SAD, which are characterized by avoidable threats, active risk assessment would lead to decreased tolerance and more experiential avoidance behaviors.

Our negative finding regarding differences between the subsamples in terms of concealing is at odds to previous studies that found a negative association between concealing and anxiety.^5,6^ We suggest that cultural factors may play a role here, with increased concealing across both Brazilian clinical samples and Brazilian healthy controls. Arguably, suppression of emotions, as assessed by the concealing scale of the ASQ, has been associated with fewer negative consequences for individuals hailing from a collectivist rather than an individualist cultural background.^23-25^

These findings may have therapeutic implications. For instance, by leading to increased acceptance and the ability to tolerate difficult emotions, Acceptance and Commitment Therapy,^26^ and mindfulness-based therapies may increase willingness to participate in distressing yet highly effective tasks, such as exposure and response prevention.^27^ Therefore, therapists could work with OCD and SAD patients with the intention of increasing openness to experiences, which could be redirected towards living a more meaningful life.

Our study has some significant limitations. First, the sample was relatively small, particularly the healthy controls group. A larger sample could have resulted in a greater ability to detect smaller differences between other affective styles. Further research should be conducted with larger sample sizes to investigate the effect of gender, other potential covariates, and affective styles other than tolerance. Additionally, it would be important to detail characteristics of the psychotherapies received, including whether they were strictly defined CBT and mindfulness, since these are known to have an impact on affective style.^28,29^ This would be particularly important for adjustment, which exhibited only a general effect, but no statistically significant differences between the groups.

Another limitation is the lack of a measure to assess transdiagnostic severity or impairment, such as the Clinical Global Impression (CGI) or Global Assessment of Functioning (GAF), to observe whether the diagnostic groups comprised participants with the same level of severity; the only scales used to observe severity were specific for each disorder, such as DOCS, PAS, and SPIN, which did not enable comparisons between groups. This may constitute bias in this research, since some groups may have had participants with lower severity, matching the control group. Additionally, the fact that assignment of participants showing more than one diagnosis of interest was based on their most clinically significant diagnosis may be considered somewhat arbitrary and a potential source of bias.

## References

[1] Hofmann SG, Kashdan TB (2010). The affective style questionnaire: development and psychometric properties. J Psychopathol Behav Assess.

[2] Thompson RA (1994). Emotion regulation: a theme in search of definition. Monogr Soc Res Child Dev.

[3] Davidson RJ (1998). Affective style and affective disorders: perspectives from affective neuroscience. Cogn Emot.

[4] Ito M, Hofmann SG (2014). Culture and affect: the factor structure of the affective style questionnaire and its relation with depression and anxiety among Japanese. BMC Res Notes.

[5] Totzeck C, Teismann T, Hofmann SG, von Brachel R, Zhang XC, Pflug V (2018). Affective styles in mood and anxiety disorders - Clinical validation of the "Affective Style Questionnaire" (ASQ). J Affect Disord.

[6] Wang J, Xu W, Fu Z, Yu W, He L, Sun L (2019). Psychometric properties of the Chinese version of the Affective Style Questionnaire and its role as a moderator of the relationship between stress and negative affect. J Health Psychol.

[7] Aldao A, Nolen-Hoeksema S, Schweizer S (2010). Emotion-regulation strategies across psychopathology: A meta-analytic review. Clin Psychol Rev.

[8] Lecrubier Y, Weiller E, Hergueta T, Amorim P, Bonora LI, Lépine JP (1992). DSM IV, tradução para o português por P Amorim.

[9] Abramowitz JS, Deacon BJ, Olatunji BO, Wheaton MG, Berman NC, Losardo D (2010). Assessment of obsessive-compulsive symptom dimensions: development and evaluation of the Dimensional Obsessive-Compulsive Scale. Psychol Assess.

[10] Bandelow B (1995). Assessing the efficacy of treatments for panic disorder and agoraphobia. II. The Panic and Agoraphobia Scale. Int Clin Psychopharmacol.

[11] Connor KM, Davidson JRT, Erik Churchill L, Sherwood A, Foa E, Weisler RH (2000). Psychometric properties of the social phobia inventory (SPIN). New self- rating scale. Br J Psychiatry.

[12] Osório FL, Crippa JAS, Loureiro SR (2010). Evaluation of the psychometric properties of the Social Phobia Inventory in university students. Compr Psychiatry.

[13] Beck AT, Epstein N, Brown G, Steer RA (1988). An inventory for measuring clinical anxiety: Psychometric properties. J Consult Clin Psychol.

[14] Quintão S, Delgado AR, Prieto G (2013). Validity study of the Beck Anxiety Inventory (Portuguese version) by the Rasch Rating Scale model. Psicol Reflex Critica.

[15] Beck AT, Ward C, Mock J M. M, Erbaugh J (1961). Beck Depression Inventory (BDI). Arch Gen Psychiatry.

[16] Gorenstein C, Andrade LHS (1996). Validation of a portuguese version of the Beck Depression Inventory and State-Trait Anxiety Inventory in Brazilian subjects. Brazilian J Med Biol Res.

[17] Hofmann SG, Sawyer AT, Fang A, Asnaani A (2012). Emotion dysregulation model of mood and anxiety disorders. Depress Anxiety.

[18] Hayes SC, Strosahl K, Wilson KG (1999). Acceptance and commitment therapy: An experiential approach to behavior change.

[19] Hayes SC, Wilson KG, Gifford E V, Follette VM, Strosahl K (1996). Experiential avoidance and behavioral disorders: A functional dimensional approach to diagnosis and treatment. J Consult Clin Psychol.

[20] Kashdan TB, Farmer AS, Adams LM, Ferssizidis P, McKnight PE, Nezlek JB (2013). Distinguishing healthy adults from people with social anxiety disorder: Evidence for the value of experiential avoidance and positive emotions in everyday social interactions. J Abnorm Psychol.

[21] Yap K, Mogan C, Moriarty A, Dowling N, Blair-West S, Gelgec C (2018). Emotion regulation difficulties in obsessive-compulsive disorder. J Clin Psychol.

[22] Gray JA, McNaughton N (2000). The neuropsychology of anxiety: an enquiry into the functions of the septo-hippocampal system.

[23] Arens EA, Balkir N, Barnow S (2013). Ethnic variation in emotion regulation. J Cross Cult Psychol.

[24] Matsumoto D, Yoo SH, Nakagawa S (2008). Culture, emotion regulation, and adjustment. J Pers Soc Psychol.

[25] Voswinckel I, Spranz S, Langguth N, Stangier U, Gawrilow C, Steil R (2019). Suppression, reappraisal, and acceptance of emotions: a comparison between Turkish immigrant and German adolescents. J Cult Cogn Sci.

[26] Hayes SC, Strosahl KD, Wilson KG (2011). Acceptance and commitment therapy: The process and practice of mindful change.

[27] Deacon BJ, Abramowitz JS (2004). Cognitive and behavioral treatments for anxiety disorders: A review of meta-analytic findings. J Clin Psychol.

[28] Graser J, Höfling V, Weßlau C, Mendes A, Stangier U (2016). Effects of a 12-week mindfulness, compassion, and loving kindness program on chronic depression: A pilot within-subjects wait-list controlled trial. J Cogn Psychother.

[29] Totzeck C, Teismann T, Hofmann SG, von Brachel R, Zhang XC, Wannemüller A (2019). Affective styles in panicdisorder and specific phobia: changes through cognitive behavior therapy and prediction of remission. Behav Ther.

